# Efficacy and safety of aldosterone synthase inhibitors for uncontrolled hypertension: a meta-analysis of randomized controlled trials and systematic review

**DOI:** 10.3389/fphar.2025.1664810

**Published:** 2025-09-22

**Authors:** Yifan Gao, Xu Mu, Xingxue Pang

**Affiliations:** ^1^ Dongzhimen Hospital, Beijing University of Chinese Medicine, Beijing, China; ^2^ Third Department of Cardiology, Dongzhimen Hospital, Beijing University of Chinese Medicine, Beijing, China

**Keywords:** uncontrolled hypertension, aldosterone synthase inhibitors, hyperkalemia, blood pressure reduction, meta-analysis

## Abstract

**Background:**

Uncontrolled hypertension is a major global health concern. Aldosterone synthase inhibitors (ASIs) show promise as a new treatment approach for blood pressure management.

**Methods:**

A systematic review and meta-analysis were conducted on randomized controlled trials comparing ASIs versus placebo for uncontrolled hypertension. The search included PubMed, Cochrane Library, Web of Science, and Embase databases from inception to 7 July 2025, limited to English-language publications. Data extraction was performed independently by two authors.

**Results:**

Four randomized controlled trials involving 1,838 patients (mean age 62 years; 47% female) were analyzed. The results demonstrated that ASIs significantly reduced office systolic blood pressure by 8.21 mmHg (95% CI, −10.64 to −5.78; P < 0.0001) and diastolic blood pressure by 3.64 mmHg (95% CI, −5.65 to −1.63; P = 0.0004). The risk ratio for adverse events was 1.42 (95% CI, 1.25-1.60; P < 0.00001), with a similar trend observed for serious adverse events (risk ratio 1.17; 95% CI, 0.63-2.17; P = 0.61). No treatment-related deaths occurred. However, ASIs were associated with a significantly higher risk of hyperkalemia (risk ratio 7.97; 95% CI, 2.27-27.99; P = 0.001).

**Conclusion:**

ASIs significantly lower blood pressure in hypertensive patients with an acceptable safety profile, though hyperkalemia risk requires monitoring. These results suggest ASIs may be a viable hypertension treatment, but larger studies are needed.

## Graphical Abstract

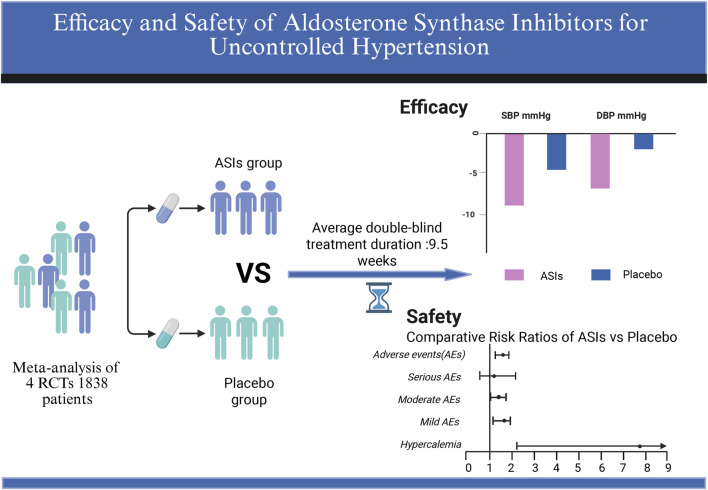

## 1 Introduction

Hypertension remains a major global health burden and a significant contributor to cardiovascular morbidity and mortality (Poulter et al., 2015). Despite the availability of various antihypertensive medications, a substantial proportion of patients fail to achieve adequate blood pressure control, a condition termed uncontrolled hypertension (Nardoianni et al., 2024; Padmanabhan et al., 2020). This therapeutic gap underscores the urgent need for novel pharmacological approaches targeting alternative pathways in blood pressure regulation.

Aldosterone excess is a key driver of uncontrolled and resistant hypertension (Bansal et al., 2025). Aldosterone synthase inhibitors (ASIs) represent a novel class of antihypertensive agents that selectively inhibit CYP11B2, the enzyme responsible for aldosterone synthesis (Marzano et al., 2025; Dogra et al., 2023; Mulatero et al., 2023). Although several Phase II trials have demonstrated the efficacy and safety of ASIs, the overall efficacy and safety profile of ASIs in patients with uncontrolled hypertension remains incompletely characterized due to the limited scope and inconsistent outcomes of these studies.

In a refractory hypertension trial, once-daily oral administration of 2 mg Baxdrostat for 12 weeks resulted in a mean systolic blood pressure (SBP) reduction of 11.0 mmHg compared to placebo (Freeman et al., 2023). Similarly, 50 mg Lorundrostat administered once daily demonstrated significant SBP-lowering effects in patients with uncontrolled hypertension (Saxena et al., 2025). These findings underscore the therapeutic potential of ASIs in managing treatment-resistant hypertension.

While a series of systematic reviews and meta-analyses have evaluated the therapeutic efficacy and safety of aldosterone synthase inhibitors (ASIs) in hypertensive patients, their conclusions remain inconsistent, and no prior analysis has specifically focused on ASIs’ safety and efficacy in uncontrolled hypertension populations (Marzano et al., 2025; Siddiqui et al., 2024). To address these knowledge gaps, our study synthesizes pooled data from multiple randomized clinical trials, elucidating the clinical value of ASIs in uncontrolled hypertension and providing evidence-based insights to guide future research and therapeutic decision-making.

## 2 Methods

The study protocol was prospectively registered in PROSPERO (CRD420251090802). In accordance with the updated PRISMA 2020 guidelines (Page et al., 2021), we designed, executed, and documented this systematic review, with the completed checklist provided in Supplementary Appendix.

### 2.1 Data sources and searches

The literature search was conducted across four major databases: PubMed, EMBASE, Cochrane Library, and Web of Science, encompassing publications from their inception through 7 July 2025. Our search strategy incorporated key terms including ASI (aldosterone synthase inhibitor), BP (blood pressure), uncontrolled hypertension, and randomized controlled trial. The PICOS framework (Population, Intervention, Comparison, Outcomes, Study design) informed our review criteria, research questions, and search methodology. For comprehensive details regarding the search strategy, please refer to the Supplementary Table S1.

### 2.2 Eligibility criteria and study selection

This systematic review included randomized controlled trials (RCTs) involving patients with uncontrolled hypertension who received aldosterone synthase inhibitors versus placebo, with outcomes assessing blood pressure changes and adverse events. The selection process involved: (1) 70 removing duplicates; (2) screening titles/abstracts to exclude non-RCTs; (3) excluding studies 71 unrelated to uncontrolled hypertension; and (4) full-text review for final eligibility. Two independent reviewers (YG and XP) performed screening, with disagreements resolved by a third reviewer (XM). Potentially eligible studies underwent full-text assessment.

### 2.3 Data extraction

The data extraction was performed independently by two authors (YG and XP) using standardized forms. The extracted data encompassed study characteristics, participant demographics, intervention details, blood pressure changes, biochemical parameters, and adverse events. All data were entered into a dedicated database and underwent independent verification. Any discrepancies were resolved through consensus-based discussions.

### 2.4 Outcomes and definitions

This study aimed to (Poulter et al., 2015) compare the effects of oral aldosterone synthase inhibitors (ASIs) versus placebo on blood pressure in patients with uncontrolled hypertension (primary objective), and (Nardoianni et al., 2024) evaluate the safety profile of ASIs (secondary objective). The primary efficacy endpoint was the change in systolic blood pressure from baseline to study endpoint, while secondary efficacy outcomes included changes in diastolic blood pressure during the same period. Safety was assessed by analyzing the frequency and severity of all spontaneously reported adverse events. Uncontrolled hypertension was defined as blood pressure ≥130/80 mmHg in patients receiving antihypertensive therapy.

### 2.5 Risk of bias and sensitivity analysis

We assessed the risk of bias in eligible trials using the updated Cochrane tool (RoB 2, version 2), which evaluates several domains: randomization process, deviations from intended interventions, missing outcome data, measurement of outcomes, and selection of reported results (Sterne et al., 2019). Guided by signaling questions, we categorized the risk of bias as low, some concerns, or high. Two independent reviewers (YG and XM) conducted these assessments.

We systematically conducted sensitivity analyses by sequentially excluding individual studies to examine their impact on the overall results.

### 2.6 Statistical analysis

We performed a descriptive analysis for each trial, with detailed results presented in Table 1; Supplementary Table S2. For continuous outcomes such as blood pressure, we evaluated the mean change from baseline to endpoint compared with placebo. For dichotomous outcomes including adverse events, we calculated the risk ratio (RR) with corresponding 95% confidence intervals (CI) for each trial.

**TABLE 1:** General characteristics of the included studies.

Trial ID	Trial design	No. of patients	Study population	Primary outcome definition	ASIs treatment	Comparator (s)	Age mean (SD), y	Male sex, n (%)	BMI, mean (SD), kg ⁄ m2	Diabetes, n (%)	Baseline BP, mmHg	eGFR, mean (SD), mL/min per 1.73 m2
Freeman 2023	Randomized, double-blind, placebo-controlled, parallel-group, dose-ranging trial	275	treatment-resistant hypertension	Office SBP change at 12 weeks	Baxdrostat	placebo	62 (11)	153 (56)	33 (5)	105 (38)	148/88	84 (19)
Laffin 2023	Randomized, double-blind, placebo-controlled, dose-ranging, multicenter Phase 2 study	200	uncontrolled hypertension	Change in office SBP at 8 weeks	Lorundrostat	placebo	66 (10)	80 (40)	31 (5)	79 (40)	142/81	80 (15)
Laffin 2025	Randomized, double-blind, placebo-controlled, multicenter Phase 2 b trial with a dose-adjustment component (50 mg with optional escalation to 100 mg)	285	uncontrolled hypertension	24 h ambulatory SBP change at 12 weeks	Lorundrostat	placebo	62 (10)	172 (60)	32 (5)	119 (42)	142/85	81 (18)
Saxena 2025	Randomized, double-blind, placebo-controlled, multicenter Phase 3 trial with a dose-adjustment component (50 mg with optional escalation to 100 mg)	1,078	uncontrolled hypertension	Change in automated office systolic BP at week 6	Lorundrostat	placebo	62 (11)	575 (53)	33 (7)	338 (32)	148/87	91 (17)

All statistical analyses were performed using RevMan, version 5.4.1, and STATA, version 17.0. (The Cochrane Collaboration) (Sun et al., 2010). Heterogeneity among studies was assessed using the Q-test. A random-effects model was used for all meta-analyses to account for potential clinical and methodological heterogeneity across trials, including differences in study populations, dosing regimens, and outcome definitions.

We conducted two sensitivity analyses to verify the robustness of our findings. First, we performed *a priori* subgroup analyses to evaluate pooled estimates across different classes of ASIs. Second, we carried out leave-one-out analyses to examine the influence of individual studies on the overall effect size and to identify any potentially influential outliers. Additionally, we assessed potential publication bias in the meta-analyses through funnel plots and Egger’s regression tests.

Where quantitative data were insufficient or showed substantial heterogeneity, we employed descriptive synthesis methods. All statistical tests were two-sided, with a significance threshold set at p ≤ 0.05.

## 3 Results

### 3.1 Study selection

A total of 832 records were identified through the initial search, from which 311 duplicate records were removed.

**FIGURE 1:**
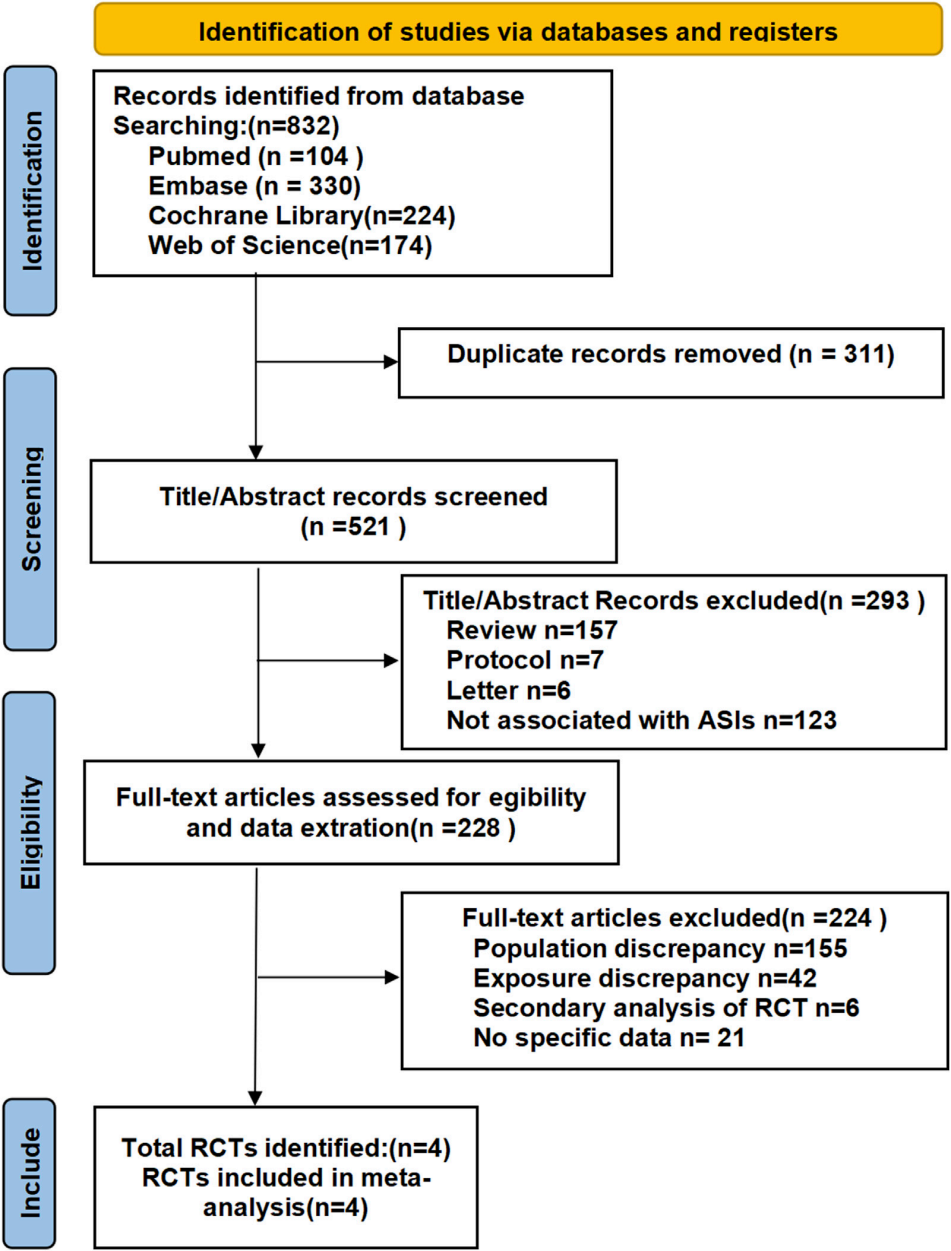
Flow chart of the article selection procedure for meta-analysis.

Following the removal of duplicates, we screened 521 records by title/abstract and excluded 293 irrelevant studies. Full-text review of 228 articles led to the exclusion of 224 records due to: ineligible populations (n = 155), non-comparable interventions (n = 42), secondary RCT analyses (n = 6), or registered trials without available data (n = 21). Four qualifying RCTs (N = 1,838 participants) were included in our analysis: one evaluating Baxdrostat monotherapy and three assessing Lorundrostat monotherapy, all versus placebo for uncontrolled hypertension (Freeman et al., 2023; Laffin et al., 2023; Laffin et al., 2025; Saxena et al., 2025). The mean double-blind treatment duration across studies was 9.5 weeks, varying from 6 to 12 weeks. The study selection process is illustrated in Figure 1.

### 3.2 Baseline characteristics

The study characteristics are summarized in Table 1; Supplementary Table S2. Our systematic review and meta-analysis included 1,838 individuals from four distinct studies. Of these, 1,368 participants (74%) were allocated to the ASI intervention group, while the remaining 470 (26%) were assigned to the placebo group.

The included participants had a mean age of 62 years, with males comprising 53% (n = 980) and females 47% (n = 858). Over 85% of participants received either ACEI inhibitors (angiotensin-converting enzyme inhibitors) or ARBs (angiotensin receptor blockers), while more than 90% were treated with diuretics. The usage rates ranged from 52% to 100% in the intervention group and 53%–100% in the control group.

### 3.3 Changes in systolic BP

**FIGURE 2:**
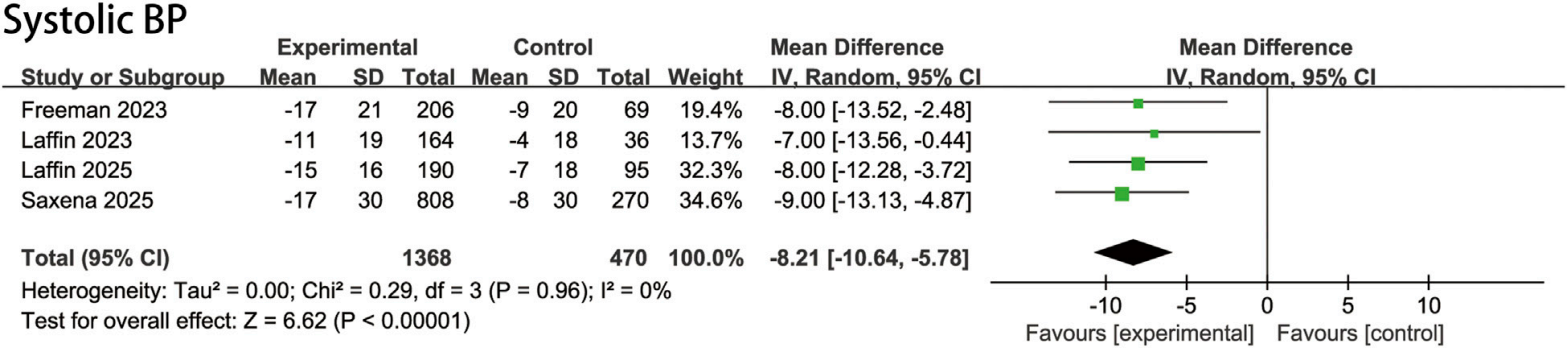
Pooled treatment effect estimates of aldosterone synthase inhibitors compared with placebo on systolic blood pressure in patients with uncontrolled hypertension, random-effects model.

The overall estimates and subgroup stratification of our primary efficacy outcomes are shown in Figure 2; Supplementary Figure S1. The ASI group had a mean baseline SBP of 146.5 mmHg, compared with 146.8 mmHg in the placebo group. The meta-analysis demonstrated a significant reduction in systolic blood pressure (SBP): the pooled mean difference in SBP change was −8.2 mmHg ([95% CI, −10.6 to −5.8 mmHg]; P < 0.00001; I^2^ = 0%), indicating a statistically significant improvement with ASI therapy.

### 3.4 Changes in diastolic BP

**FIGURE 3:**
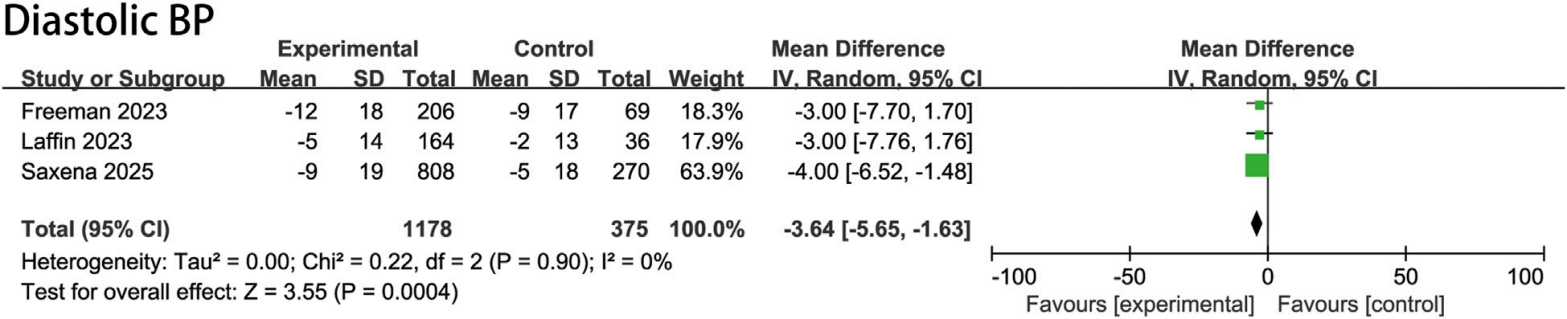
Pooled treatment effect estimates of aldosterone synthase inhibitors compared with placebo on diastolic blood pressure in patients with uncontrolled hypertension, random-effects model.

The ASI group had a mean baseline DBP of 86.4 mmHg, identical to the placebo group’s 86.4 mmHg. Pooled analysis demonstrated a DBP reduction of −3.6 mmHg (95% CI, −5.7 to −1.6 mmHg; P = 0.0004; I^2^ = 0%) compared with the placebo group (Figure 3).

### 3.5 Adverse events and tolerability

**FIGURE 4:**
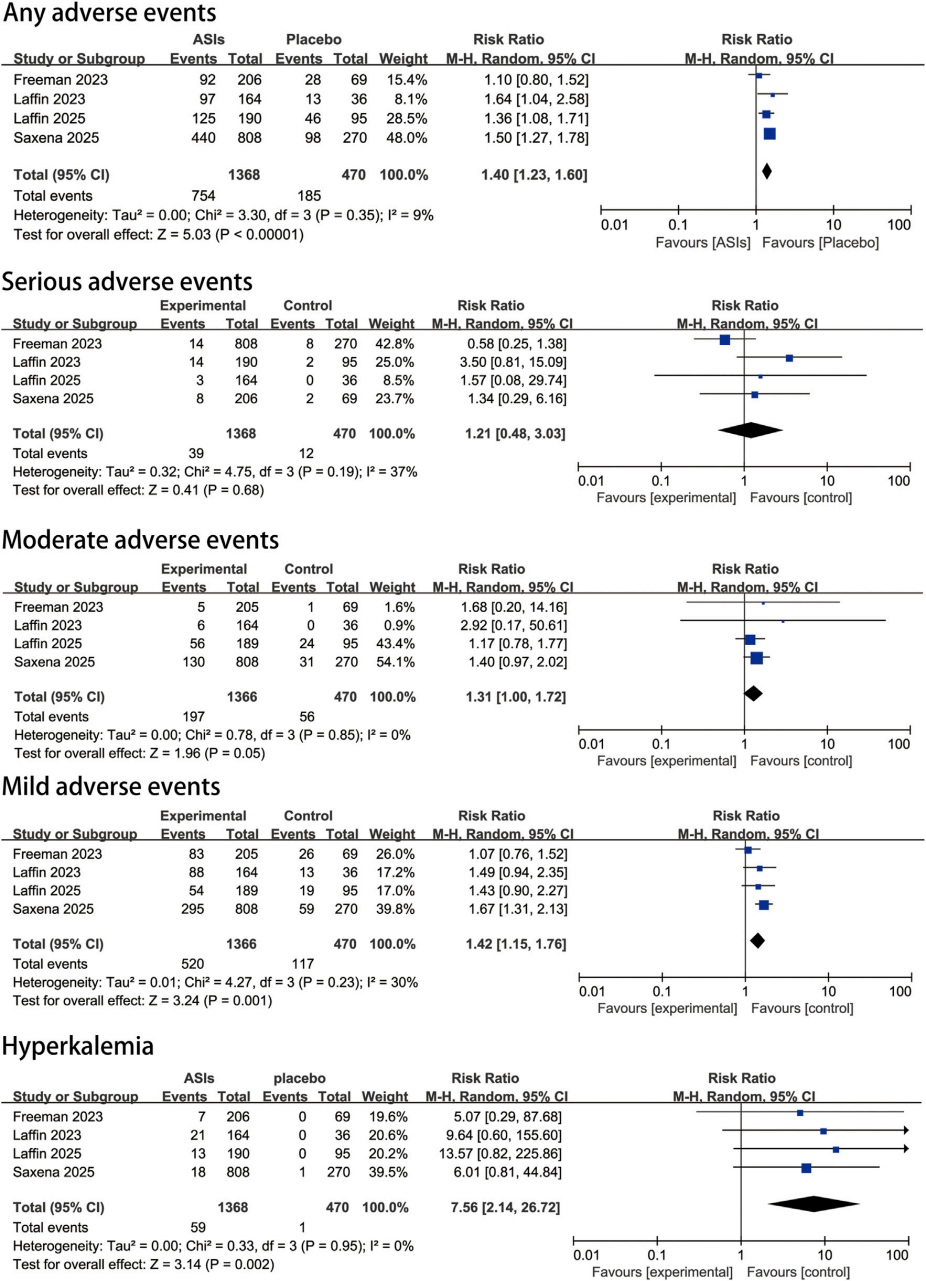
Pooled safety effect estimates of aldosterone synthase inhibitors compared with placebo in patients with uncontrolled hypertension, random-effects model.

The pooled estimates of adverse events and tolerability, including safety outcomes in both overall and specific subgroups, are presented in Figure 4. Our analysis showed that compared with placebo, ASIs were not associated with a significant increase in serious adverse events (RR, 1.2 [95% CI, 0.5–3.0]; P = 0.19; I^2^ = 37%). However, the risk of overall adverse events was significantly higher in the ASI group (RR, 1.4 [95% CI, 1.2–1.6]; P < 0.00001; I^2^ = 9%). Specifically, the risk of moderate adverse events was higher with ASIs (RR, 1.3 [95% CI, 1.0–1.7]; P = 0.05; I^2^ = 0%), and the risk of mild adverse events was also higher in the ASI group (RR, 1.4 [95% CI, 1.2–1.8]; P = 0.001; I^2^ = 30%). The risk of hyperkalemia was markedly elevated compared to placebo (RR, 7.6 [95% CI, 2.1–26.7]; P = 0.002; I^2^ = 0%). Nevertheless, the mean increase in plasma potassium levels was minimal (0.39 mmol/L in the ASI group versus 0.02 mmol/L in the placebo group). Only one death occurred, which was deemed unrelated to treatment, indicating that ASIs were generally well tolerated.

### 3.6 Subgroup analysis and sensitivity analysis

Our subgroup analysis, stratified by specific ASIs (Baxdrostat and Lorundrostat), confirmed the efficacy of ASIs on systolic blood pressure of ASIs compared with placebo. No significant differences were observed between subgroups, with minimal statistical heterogeneity (Supplementary Figure S1). Both sensitivity analyses supported these findings (Supplementary Figures S1, S2).

First, the *a priori* subgroup sensitivity analysis demonstrated consistent efficacy and safety outcomes across different ASI types (Supplementary Figure S1). Second, the leave-one-out sensitivity analysis showed that excluding individual studies did not significantly alter the overall efficacy and safety outcomes of ASIs relative to placebo (Supplementary Figure S2).

### 3.7 Publication bias

We rigorously assessed publication bias using Egger’s test and funnel plots, which demonstrated no significant bias in studies examining the mean difference in systolic blood pressure changes and adverse events with ASI therapy compared to placebo (Supplementary Figure S3). However, potential bias was detected in studies analyzing the mean difference in diastolic blood pressure changes.

### 3.8 Study quality

The quality assessment of included studies (Supplementary Figure S4) demonstrated a consistently low risk of bias across all critical methodological domains, including randomization procedures, allocation concealment, intervention adherence, completeness of outcome data, accuracy of outcome measurement, and reporting transparency. This rigorous methodological approach throughout the trial implementation significantly enhances the reliability and validity of our study findings.

## 4 Discussion

### 4.1 Main findings and interpretations

Our findings demonstrate that patients with uncontrolled hypertension can benefit substantially from ASI therapy. The meta-analysis showed significant reductions in both systolic and diastolic blood pressure with generally good tolerability, supporting ASIs as a promising therapeutic strategy for hypertension management.

Mechanistically, targeted inhibition of aldosterone synthesis represents a distinct approach compared with existing antihypertensive classes, particularly advantageous for patients with aldosterone-excess phenotypes (Bianchi et al., 2019; Pu et al., 2025; Hung et al., 2025; Miura et al., 2025; Pitt and Williams, 2024). This rationale is supported by recent trials in treatment-resistant cohorts, which reported 8–12 mmHg reductions in systolic blood pressure (Hargovan and Ferro, 2014).

A key safety consideration is the risk of hyperkalemia, which results from impaired renal potassium excretion secondary to aldosterone suppression. This risk is amplified in patients with chronic kidney disease, where potassium handling is already compromised (Marzano et al., 2025; Saxena et al., 2025; Pu et al., 2025). Careful monitoring is therefore essential to optimize outcomes in high-risk populations.

### 4.2 How can patients with uncontrolled hypertension benefit from ASIs

#### 4.2.1 Direct antihypertensive effects

ASIs may more effectively suppress excessive aldosterone activation in patients with suboptimally controlled hypertension, thereby optimizing blood pressure management (Hung et al., 2025). By selectively inhibiting CYP11B2, ASIs reduce aldosterone production and directly lower blood pressure (Azizi et al., 2025). Unlike mineralocorticoid receptor antagonists (MRAs), which block receptor binding, ASIs act upstream to directly suppress aldosterone synthesis, potentially providing more comprehensive regulation of the renin–angiotensin–aldosterone system (RAAS) while avoiding sex hormone–related adverse effects associated with MRAs (Mazzieri et al., 2024; Namsolleck and Unger, 2014).

#### 4.2.2 Broader therapeutic potential beyond blood pressure control

Beyond their antihypertensive effects, ASIs may provide additional clinical benefits in conditions where aldosterone plays a key pathogenic role (He et al., 2025).

In chronic kidney disease (CKD), aldosterone contributes to glomerulosclerosis, tubulointerstitial fibrosis, and proteinuria through pro-inflammatory and pro-fibrotic mechanisms (Munoz-Durango et al., 2016; Theodorakopoulou et al., 2025). Preclinical studies and early clinical evidence suggest that selective inhibition of aldosterone synthesis with ASIs can attenuate renal fibrosis and reduce proteinuria, thereby providing renoprotective effects independent of blood pressure control (Cunningham and Lam, 2025; Ferreira et al., 2025; Tuttle et al., 2024).

In addition to renal protection, aldosterone has also been implicated in metabolic dysregulation, including insulin resistance and impaired glucose homeostasis. Elevated aldosterone levels are associated with an increased risk of type 2 diabetes mellitus (DM) and metabolic syndrome (Bansal et al., 2025). By reducing aldosterone production, ASIs may improve insulin sensitivity and exert favorable effects on lipid metabolism, highlighting their potential application in patients with DM and related metabolic disorders (Ferreira et al., 2025; Gomez-Sanchez and Gomez-Sanchez, 2023).

In the endocrine field, ASIs hold promise for the management of disorders characterized by aldosterone excess, such as primary aldosteronism (Gomez-Sanchez and Gomez-Sanchez, 2023). Unlike MRAs, ASIs directly inhibit aldosterone biosynthesis without interfering with androgen or progesterone receptors, and recent clinical trials, such as BrigHTN, demonstrated blood pressure reduction without suppression of cortisol synthesis. This pharmacological selectivity may translate into improved safety and tolerability compared with MRAs (Freeman et al., 2023).

While these findings are promising, further validation from large-scale, long-term randomized trials is required to establish the broader therapeutic role of ASIs beyond hypertension (Ferreira et al., 2025).

#### 4.2.3 ASIs may enhance efficacy when combined with other antihypertensive agents

In addition to monotherapy, ASIs hold promise as part of combination regimens for resistant hypertension (Mancia et al., 2019). Current treatment algorithms typically include a renin–angiotensin system blocker, a calcium-channel blocker, and a thiazide-like diuretic, with MRAs often used as fourth-line therapy. By acting upstream of aldosterone, ASIs provide a complementary mechanism that may overcome limitations of MRAs, such as aldosterone escape and off-target effects, thereby enhancing efficacy when integrated into multidrug treatment strategies.

### 4.3 Relationship with previous studies

The focus of this study is to evaluate the efficacy and safety of aldosterone synthase inhibitors (ASIs) in patients with uncontrolled hypertension. Uncontrolled hypertension typically refers to a condition where blood pressure remains above target levels despite treatment (Padmanabhan et al., 2020; Faconti et al., 2025). In contrast, the study by Marzano, Luigi et al. Marzano Luigi et al.’ study included a broader group of hypertensive patients, potentially including both controlled and uncontrolled hypertension patients (Marzano et al., 2025). This difference in inclusion criteria is key to explaining the differences in the results of the two studies.

Marzano Luigi et al.'s meta-analysis showed that ASIs reduced office systolic blood pressure by 6.3 mmHg (95% CI, −8.8 to −3.8; P < 0.0001) and diastolic blood pressure by 2.2 mmHg (95% CI, −4.2 to −0.2; P = 0.03). In contrast, in this study, the blood pressure-lowering effect of ASIs in patients with uncontrolled hypertension was more significant, with office systolic blood pressure reduced by 8.21 mmHg (95% CI, −10.64 to −5.78; P < 0.0001) and diastolic blood pressure reduced by 3.64 mmHg (95% CI, −5.65 to −1.63; P = 0.0004). This difference may reflect the greater therapeutic potential of ASIs in patients with uncontrolled hypertension. Patients with uncontrolled hypertension—particularly those with obesity-related hyperaldosteronism, low-renin states, or elevated baseline aldosterone—are likely to benefit more from ASIs due to their targeted inhibition of sodium retention, vascular remodeling, and sympathetic activation, potentially leading to greater blood pressure reduction.

In terms of safety, Marzano Luigi et al.'s study showed a risk ratio for adverse events of 1.1 (95% CI, 0.9–1.2; P = 0.3) and a risk ratio for serious adverse events of 1.0 (95% CI, 0.5–2.3; P = 0.95). In this study, the risk ratio for adverse events was 1.40 (95% CI, 1.23-1.60; P < 0.00001), which was slightly higher than that of Marzano Luigi et al.'s study. The risk of hyperkalemia was also higher in this study, with a risk ratio of 7.56 (95% CI, 2.14-26.72; P = 0.002), while in Marzano Luigi et al.'s study it was 2.5 (95% CI, [1.2–5.4]; P < 0.02). This may be related to the fact that patients with uncontrolled hypertension are often accompanied by more severe renal impairment or other metabolic disorders, making them more prone to hyperkalemia (Ritz and Pitt, 2013). Therefore, when using ASIs in patients with uncontrolled hypertension, blood potassium levels need to be monitored more closely. Across all included randomized controlled trials, hyperkalemia was typically managed through monitoring, dose adjustment, or treatment discontinuation based on its severity.

Our findings should be interpreted in the context of the existing randomized controlled trials. So far, four RCTs have been conducted, each with distinct strengths and limitations, providing further insights into the clinical profile of aldosterone synthase inhibitors (ASIs), including Lorundrostat and Baxdrostat (Freeman et al., 2023; Saxena et al., 2025; Laffin et al., 2023; Laffin et al., 2025). The TARGET-HTN trial systematically evaluated multiple doses of Lorundrostat (12.5–100 mg), establishing a dose–response relationship and providing a rationale for subsequent dose selection. The inclusion of patients with obesity and low-renin states enhanced clinical relevance, although its modest sample size (n = 200), U.S.-based cohort, and short follow-up (8 weeks) limited its generalizability and precluded long-term assessment. Building on this, the ADVANCE-HTN trial employed 24-h ambulatory blood pressure monitoring as the primary endpoint and recruited a larger, racially diverse cohort (n = 285, ∼50% Black participants), thereby improving external validity. Nevertheless, the standardized run-in phase reduced real-world representativeness, hyperkalemia occurred more frequently with Lorundrostat, and the 12-week follow-up remained insufficient to assess cardiorenal outcomes. The LAUNCH-HTN trial, the largest phase 2 study published to date (n = 1,083 across 13 countries), demonstrated a significant reduction in systolic blood pressure (−16.9 mmHg vs. −7.9 mmHg with placebo) and good short-term tolerability in patients with resistant hypertension. However, it still focused exclusively on short-term endpoints, and electrolyte disturbances, although infrequent, occurred more often than with placebo, underscoring the need for careful monitoring. Finally, the BrigHTN trial evaluated Baxdrostat, showing a dose-dependent reduction in systolic blood pressure (up to −20.3 mmHg at 2 mg) without clinically relevant effects on cortisol synthesis, suggesting potential advantages in terms of safety and selectivity. Yet, its moderate sample size (n = 248), limited racial diversity, short follow-up, and small number of hyperkalemia cases highlighted the need for further evaluation in larger and longer-term studies.

### 4.4 Limitations

Several limitations should be noted. The generalizability of our findings should be interpreted with caution. Most participants in the included trials were middle-aged to older adults with preserved renal function, and patients at the highest risk for hyperkalemia—such as those with advanced chronic kidney disease, diabetes mellitus, or advanced age—were either underrepresented or excluded due to trial eligibility criteria. Consequently, while ASIs demonstrated consistent efficacy and an acceptable safety profile in the studied populations, their applicability to these higher-risk groups remains uncertain.

The number of trials included is relatively small, which may affect the statistical power of the meta-analysis. In addition, there may be differences between trials in patient characteristics, ASI dose, and treatment duration, which may increase the heterogeneity of the study results. Future studies should include larger sample sizes, longer follow-up periods, and focus on the efficacy and safety of ASIs in different patient subgroups.

In summary, this study highlights the potential benefits and risks of using ASIs in patients with uncontrolled hypertension. Although ASIs can significantly reduce blood pressure, the risk of hyperkalemia needs to be closely monitored. Larger and longer-term studies are required to confirm their efficacy and safety, particularly with respect to cardiovascular morbidity and mortality. The observed increased risk of hyperkalemia highlights the need for careful patient selection and routine potassium monitoring. Future large-scale phase III trials with extended follow-up will be essential to establish the clinical utility of ASIs, not only in blood pressure reduction but also in their potential impact on cardiovascular and renal outcomes.

### 4.5 Prospect of further studies

Further studies are needed to fully elucidate the potential of aldosterone synthase inhibitors (ASIs) in managing hypertension and related cardiovascular diseases. Future research should focus on optimizing treatment regimens by exploring optimal dosages and combination therapies with other antihypertensive drugs, such as ACE inhibitors, angiotensin II receptor blockers, diuretics, and calcium channel blockers. Efficacy and safety should be evaluated in specific patient populations, including those with salt-sensitive hypertension, resistant hypertension, and hypertension complicated by diabetes or kidney disease. Long-term clinical trials are essential to assess the sustained impact of ASIs on cardiovascular events, renal function, and metabolic parameters, while monitoring adverse reaction rates. Investigating the non-genomic effects of aldosterone and the influence of ASIs on these effects could provide a deeper understanding of their mechanisms. Furthermore, exploring the interactions between ASIs and other endocrine systems, such as the renin-angiotensin system (RAS), sympathetic nervous system, and other endocrine pathways, will offer a more comprehensive view of their action.

## 5 Conclusion

Our systematic review and meta-analysis provide evidence that ASIs can effectively lower both systolic and diastolic blood pressure in patients with uncontrolled hypertension. By inhibiting aldosterone synthesis, ASIs offer a novel mechanism for blood-pressure control. However, the increased risk of hyperkalaemia and adverse events necessitates careful patient monitoring. Although our findings suggest a potential benefit of ASIs in managing uncontrolled hypertension, they should be interpreted cautiously given the limited number of trials and their preliminary nature. These data support further clinical development and investigation of ASIs, but larger, long-term studies are required to confirm their efficacy and safety—particularly with respect to cardiovascular morbidity and mortality. ASIs represent a promising therapeutic option, yet their use should still be regarded as experimental until more robust evidence becomes available. Future research must address these gaps and provide a clearer understanding of the role ASIs can play in clinical practice.

## Data Availability

The original contributions presented in the study are included in the article/Supplementary Material, further inquiries can be directed to the corresponding author.
