# Supplementary material for: Efficacy and safety of aldosterone synthase inhibitors for uncontrolled hypertension: a meta-analysis of randomized controlled trials and systematic review
Source: Front Pharmacol. 2025 Sep 22;16:1664810. doi: 10.3389/fphar.2025.1664810 (PMC12497756; doi:10.3389/fphar.2025.1664810)

Supplementary Material

Efficacy and Safety of Aldosterone Synthase Inhibitors for Uncontrolled Hypertension: A Meta-Analysis of Randomized Controlled Trials and Systematic Review

YiFan Gao[1], Xu Mu[1], Xingxue Pang*[1,2]

[1]Dongzhimen Hospital, Beijing University of Chinese Medicine, Beijing, China

[2]Third Department of Cardiology, Dongzhimen Hospital, Beijing University of Chinese Medicine, Beijing, China.

*** Correspondence:**
Xingxue Pang

pangxxbj@163.com

1 Supplementary Tables

1.1 Supplementary Table S1: Search Strategies

**Pubmed**<br><br>**N=104**	(("Aldosterone Synthase Inhibitors"[Mesh] OR "Aldosterone Synthase"[Mesh] AND "Enzyme Inhibitors"[Mesh]) OR (aldosterone synthase inhibitor*[tiab] OR CYP11B2 inhibitor*[tiab] OR ASI[tiab] OR LCZ696[tiab] OR baxdrostat[tiab] OR lorundrostat[tiab] OR BI 689648[tiab] OR RO6836191[tiab] OR SPI-62[tiab]))AND("Hypertension"[Mesh] OR "Resistant Hypertension"[Mesh] OR hypertens*[tiab] OR "high blood pressure"[tiab] OR "uncontrolled hypertension"[tiab] OR "treatment-resistant hypertension"[tiab] OR "refractory hypertension"[tiab])AND("Randomized Controlled Trial"[Publication Type] OR "Clinical Trial, Phase III"[Publication Type] OR "Controlled Clinical Trial"[Publication Type] OR randomized[tiab] OR randomised[tiab] OR randomization[tiab] OR randomisation[tiab] OR placebo[tiab] OR "clinical trial"[tiab] OR "clinical trials"[tiab] OR "randomly allocated"[tiab] OR "allocated randomly"[tiab] OR "controlled trial"[tiab] OR RCT[tiab] OR RCTs[tiab])Supplementary Figures and Tables
**Embase**<br><br>**N=330**	('aldosterone synthase inhibitor'/exp OR 'cyp11b2 inhibitor'/exp OR 'aldosterone synthase'/exp AND 'enzyme inhibitor'/exp OR 'aldosterone synthase inhibitor*':ti,ab OR 'cyp11b2 inhibitor*':ti,ab OR asi:ti,ab OR

	lcz696:ti,ab OR baxdrostat:ti,ab OR lorundrostat:ti,ab OR 'bi 689648':ti,ab OR 'ro6836191':ti,ab OR 'spi-62':ti,ab)AND('hypertension'/exp OR 'resistant hypertension'/exp OR hypertens*:ti,ab OR 'high blood pressure':ti,ab OR 'uncontrolled hypertension':ti,ab OR 'treatment-resistant hypertension':ti,ab OR 'refractory hypertension':ti,ab)AND('randomized controlled trial'/exp OR 'phase 3 clinical trial'/exp OR 'controlled clinical trial'/exp OR randomi?ed:ti,ab OR randomi?ation:ti,ab OR placebo:ti,ab OR 'clinical trial':ti,ab OR 'clinical trials':ti,ab OR 'randomly allocated':ti,ab OR 'allocated randomly':ti,ab OR 'controlled trial':ti,ab OR rct:ti,ab OR rcts:ti,ab)
**Cochrane Library N=224**	#1 (aldosterone synthase inhibitor* or alsin* or lorundrostat or CYP11B2 inhibitor* or ASI or LCZ696 or baxdrostat or BI 689648 or RO6836191 or SPI-62)<br><br>#2 (hypertension OR high blood pressure OR essential hypertension OR uncontrolled hypertension OR resistant hypertension)<br><br>#3 (Randomized Controlled Trial OR Clinical Trial, Phase III OR Controlled Clinical Trial OR randomized OR randomised OR randomization OR randomisation OR placebo OR clinical trial OR clinical trials OR randomly allocated OR allocated randomly OR controlled trial OR RCT OR RCTs)<br><br>#4 #1 AND #2 AND #3
**Web of Science N=174**	TS=("aldosterone synthase inhibitor*" OR "CYP11B2 inhibitor*" OR ASI OR LCZ696 OR baxdrostat OR lorundrostat OR "BI 689648" OR RO6836191 OR "SPI-62")AND TS=(hypertens* OR "high blood pressure" OR "uncontrolled hypertension" OR "treatment-resistant hypertension" OR "refractory hypertension")AND TS=(randomi?ed OR randomi?ation OR placebo OR "clinical trial*" OR "randomly allocated" OR "allocated randomly" OR "controlled trial" OR RCT OR RCTs)

1.2 Supplementary Table S2: Additional study characteristics

Trial ID	Duration of study	Trial type	No of centers	Diagnostic criteria	ASIs treatment	2 Background BP medications/⩾3 Background BP medications, n (%)	Diuretic/ACEI or ARB, n (%)
Freeman 2023	Up to 8 weeks of screening period followed by a 2-week single-blind placebo run-in phase, 12-week double-blind treatment period, and a follow-up occurred 1 week after the last dose	Multicenter	NA	Mean seated blood pressure of at least 130/80 mmHg while receiving stable doses of at least three antihypertensive medications, one of which was a diuretic	Baxdrostat, 0.5 or 1 or 2 mg QD	0(0)/273(99)	275(100)/256(93)
Laffin 2023	Up to 4 weeks for pre-screening, screening, and placebo run-in, followed by an	Multicenter	43	Systolic automated office blood pressure of 130 mm Hg or greater while	Lorundrostat, 12.5 or 50 or 100 mg QD or 12.5 or 25 mg BID	110(55)/90(45)	116(58)/162(81)

	8-week double-blind treatment phase, and a 4-week follow-up period.			taking 2 or more antihypertensive medications for at least 4 weeks at maximally tolerated doses			
Laffin 2025	Up to 3 weeks of screening and standardized antihypertensive regimen run-in period, followed by a single-blind placebo run-in period, and then a 12-week double-blind randomized treatment phase, and a follow-up occurred 4 weeks after the last dose.	Multicenter	103	Office blood pressure of at least 140/90 mmHg while taking 2 to 5 antihypertensive medications, with an average 24-hour ambulatory blood pressure of ⩾130/80 mmHg after completing a 3-week standardized antihypertensive regimen.	Lorundrostat 50 mg QD or Lorundrostat 50 mg QD, increased to 100 mg QD at week 4 if systolic BP ⩾ 130 mmHg	179(63)/106(27)	NA/NA
Saxena 2025	A screening period, followed by a	Multicenter	159	Systolic automated office blood	Lorundrostat 50 mg QD or Lorundrostat	432(40)/651(60)	1038(96)/929(86)

	2-week single-blind placebo run-in phase, then a 12-week double-blind randomized treatment phase, and a 2-week safety follow-up after the last dose			pressure of 135–180 mmHg with diastolic blood pressure of 65–110 mmHg, or isolated diastolic automated office blood pressure of 90–110 mmHg, while taking stable doses of 2–5 prescribed antihypertensive medications.	50 mg QD increased to 100 mg QD at 6 weeks if systolic BP≥ 130 mmHg, serum K^+ ≤4.8 mmol/L, serum Na^+ ≥ 135 mmol/L, eGFR >45 mL/min/1.73 m^2, <25% eGFR reduction		

For more information on Supplementary Material and for details on the different file types accepted, please see here.

2 Supplementary Figures

2.1 Supplementary Figure S1: Subgroup analysis on systolic blood pressure, stratified by specific ASIs

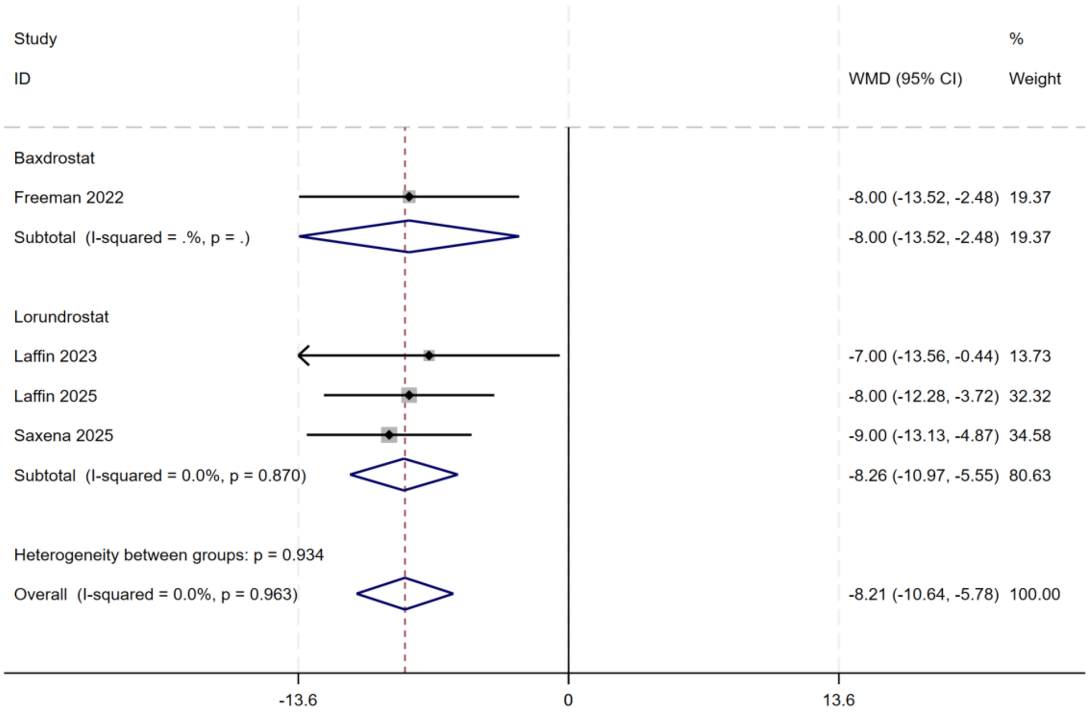

2.2 Supplementary Figure S2: Leave-One-Out Meta Analysis Results

Systolic blood pressure

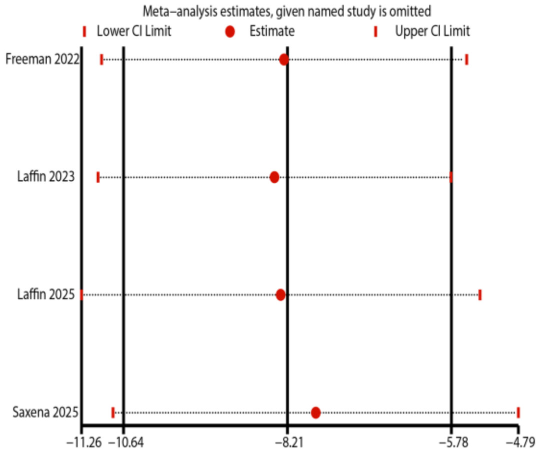

Diastolic blood pressure

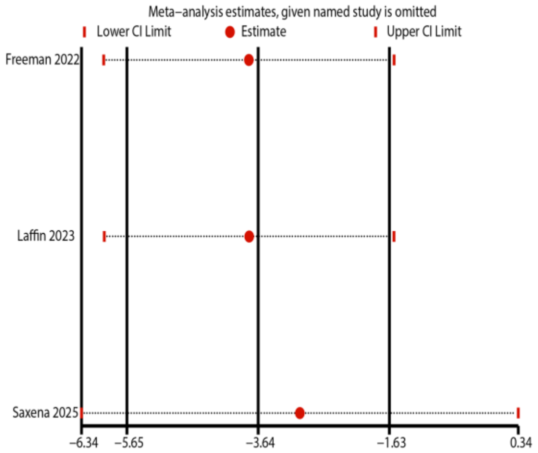

Adverse events

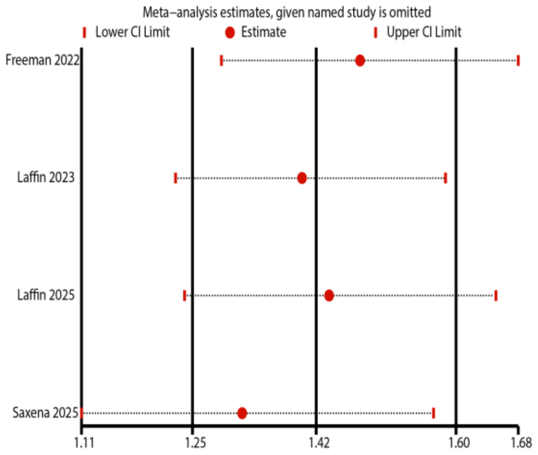

Serious adverse events

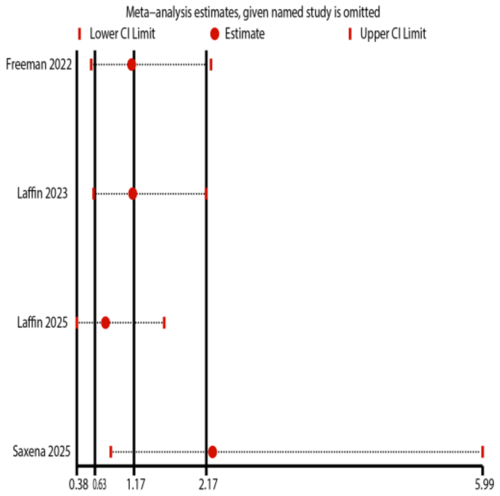

Hyperkalemia

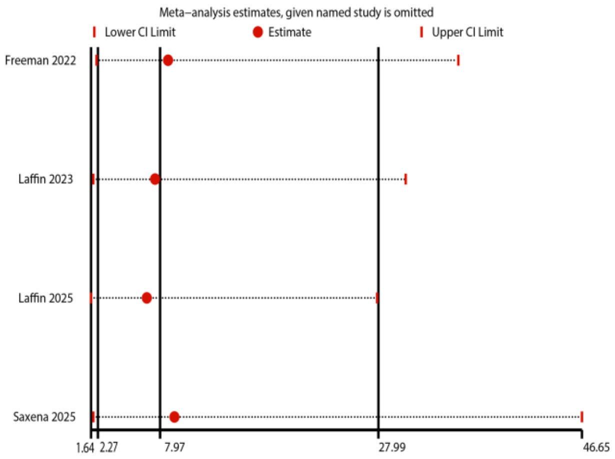

2.3 Supplementary Figure S3: Publication Bias

Systolic blood pressure

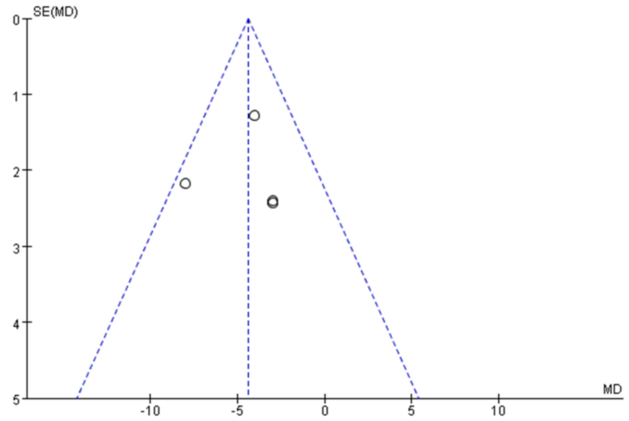

Serious adverse events

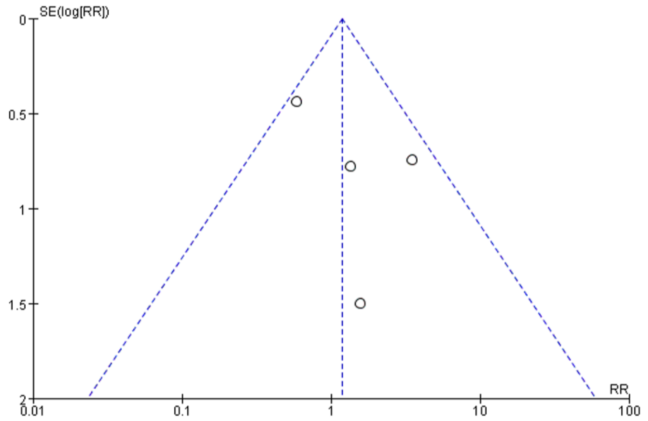

Diastolic blood pressure

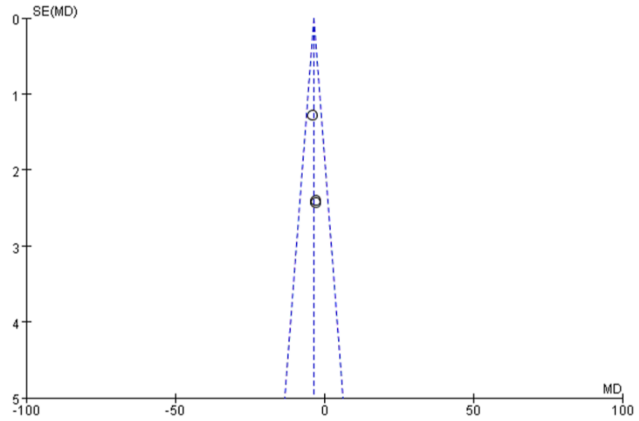

Hyperkalemia

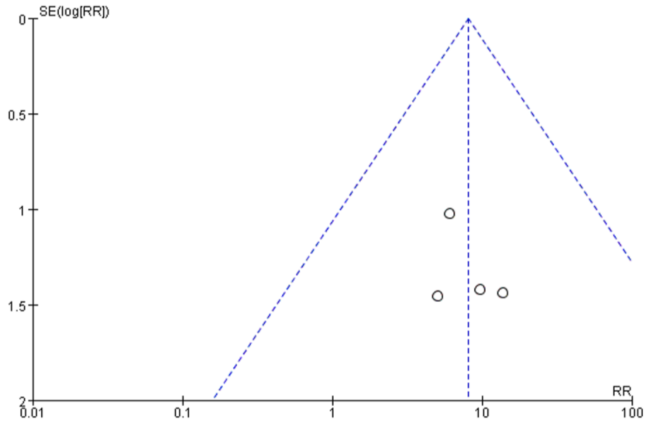

Adverse events

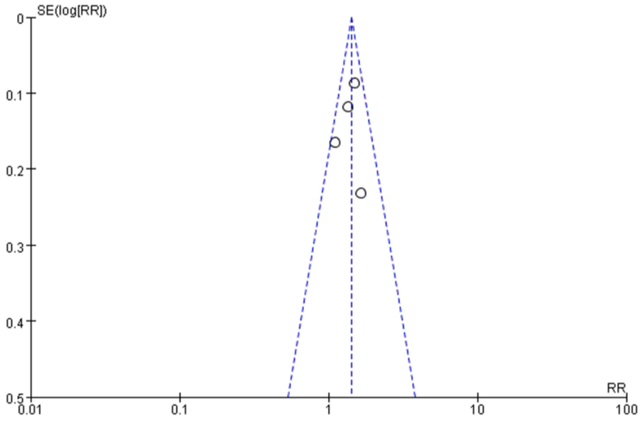

2.4 Supplementary Figure S4: Bias Risk Quality Assessment in Included Trials

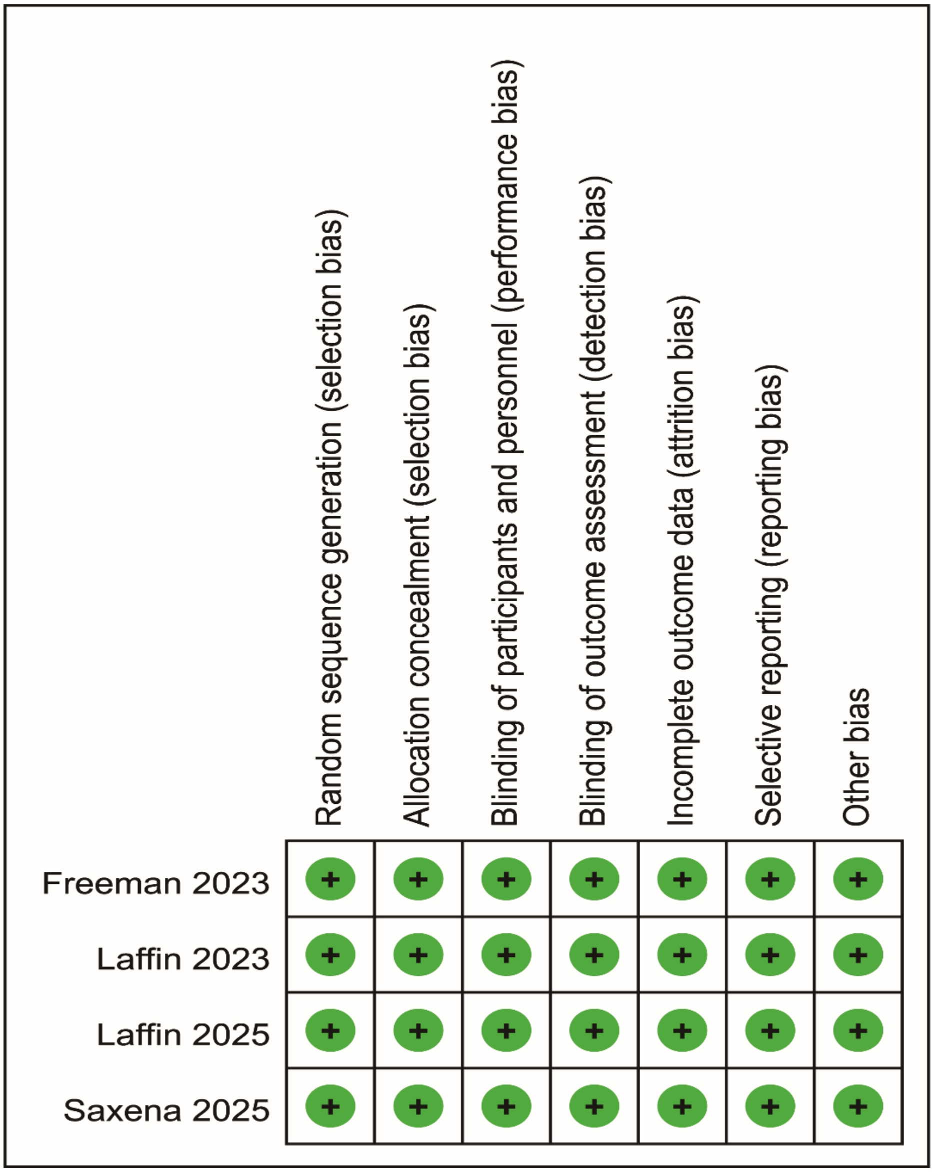

Supplement: Supplementary file 2 [file DataSheet1.pdf]
